# Supplementary material for: Measuring robust functional connectivity from resting-state MEG using amplitude and entropy correlation across frequency bands and temporal scales
Source: Neuroimage. 2021 Feb 1;226:117551. doi: 10.1016/j.neuroimage.2020.117551 (PMC7836237; doi:10.1016/j.neuroimage.2020.117551)
Supplement: Supplementary Data S1 — Supplementary Raw Research Data. This is open data under the CC BY license http://creativecommons.org/licenses/by/4.0/ [file mmc1.pdf]

Supplementary material: Measuring robust functional connectivity from resting-state MEG using amplitude and entropy correlation across frequency-bands and temporal scales

Megan Godfrey, Krish D. Singh

CUBRIC, School of Psychology, Cardiff University, Cardiff, UK

Abstract

Supplementary figures to complement Figure 6 in the main article. The relationship between MRVE time-courses and oscillatory amplitude is shown here across the whole brain, including all frequency bands and MRVE scale frequencies used in the connectivity analysis.

S	f_S (Hz)	δ	θ	α	β	γ_{40-60}
1	150.0					
2	75.0					
3	50.0					
4	37.5					
5	30.0					
6	25.0					
7	21.4					
8	18.8					
9	16.7					
10	15.0					

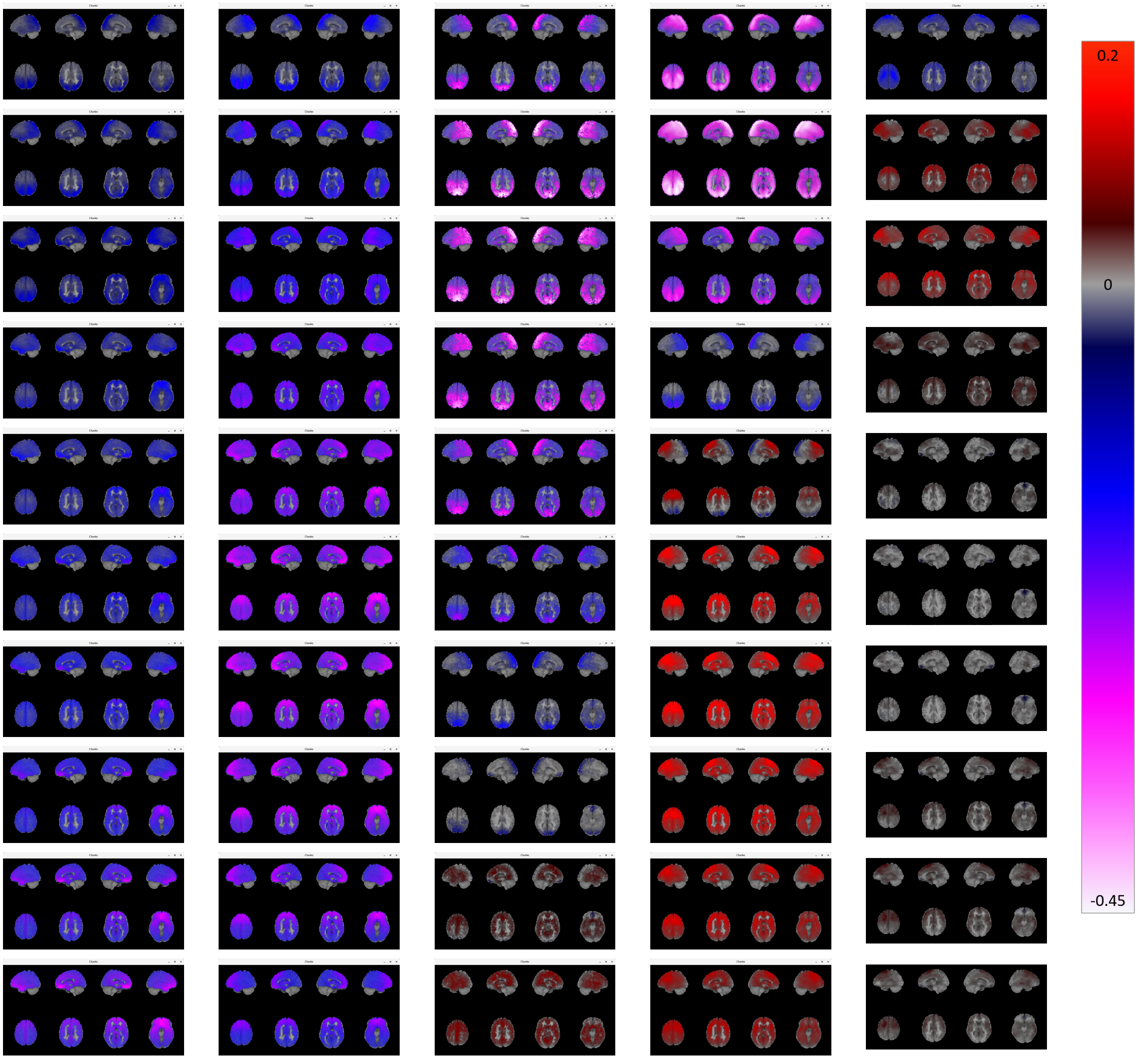

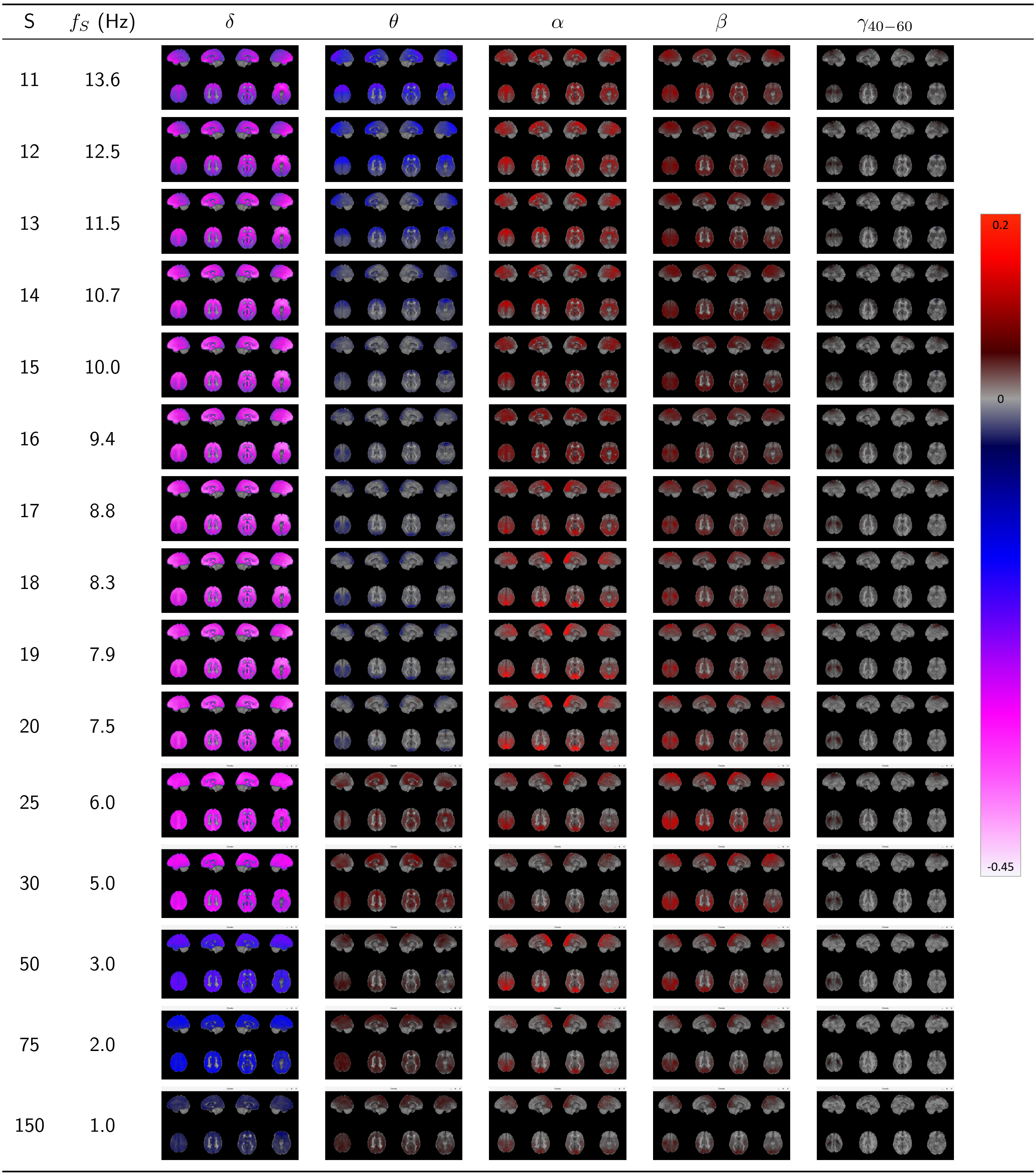

Figure 1: The whole-brain correlation between MRVE time-courses and oscillatory amplitude envelopes for scale frequencies f_S = 1-150Hz and frequency bands 1-4Hz (δ), 3-8Hz (θ), 8-13Hz (α), 13-30Hz (β) and 40-60Hz (γ_{40-60}). Correlation was found at each voxel for each participant and transformed to a z-score by applying the Fisher transformation. The 95% confidence interval was found for the z-scores calculated across all participants for each voxel. Average Pearson correlation values were found at each voxel where $z = 0$ lay outside of this confidence interval and displayed on a template brain as indicated by the colour bar.

S	f_S (Hz)	γ_{60-80}	γ_{80-100}	$\gamma_{100-120}$	$\gamma_{120-140}$	$\gamma_{140-160}$
1	150.0					
2	75.0					
3	50.0					
4	37.5					
5	30.0					
6	25.0					
7	21.4					
8	18.8					
9	16.7					
10	15.0					
11	13.6					
12	12.5					
13	11.5					
14	10.7					
15	10.0					

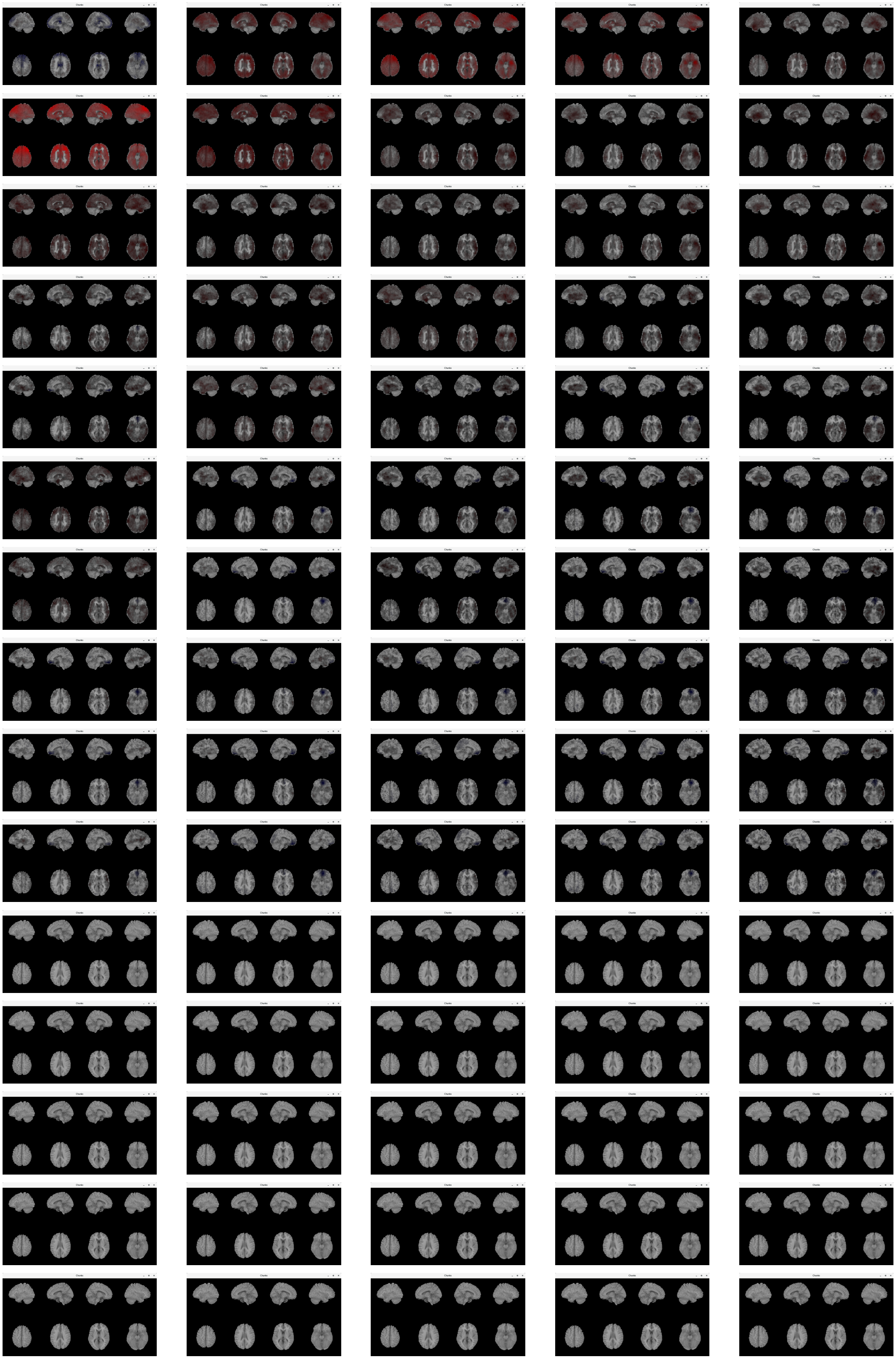

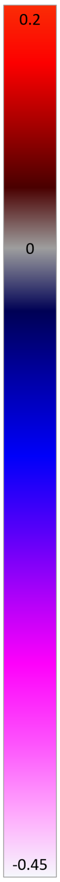

S	f_S (Hz)
16	9.4
17	8.8
18	8.3
19	7.9
20	7.5
25	6.0
30	5.0
50	3.0
75	2.0
150	1.0

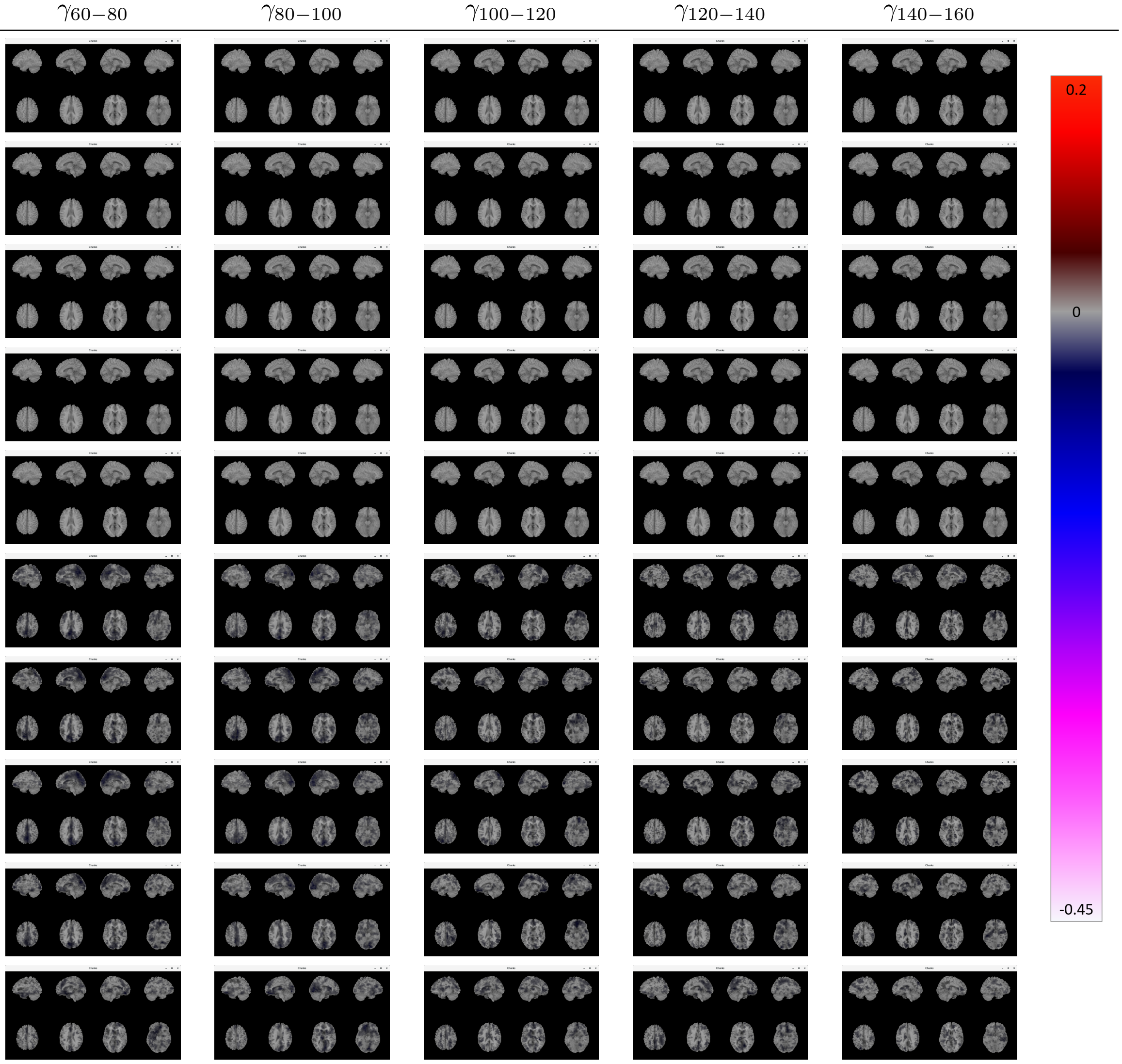

Figure 2: The whole-brain correlation between MRVE time-courses and oscillatory amplitude envelopes, for scale frequencies f_S = 1-150Hz and frequency bands 60-80Hz (γ_{60-80}), 80-100Hz (γ_{80-100}), 100-120Hz ($\gamma_{100-120}$), 120-140Hz ($\gamma_{120-140}$) and 140-160Hz ($\gamma_{140-160}$). Average correlation values over subjects are displayed on a template brain where significant, as above.
